# Editorial: Eradicating polio in Pakistan and other countries in the Eastern Mediterranean region: epidemiology, surveillance, and immunization

**DOI:** 10.3389/fpubh.2026.1903558

**Published:** 2026-09-03

**Authors:** Chukwuma Mbaeyi, Nishant Kishore, Tasha Stehling-Ariza, Sajid Soofi

**Affiliations:** 1 Global Immunization Division, Centers for Disease Control and Prevention, Atlanta, GA, United States; 2 The Carter Center, Atlanta, GA, United States; 3 Department of Pediatrics and Child Health, The Aga Khan University, Karachi, Pakistan

**Keywords:** AFP surveillance, Pakistan, polio eradication, poliovirus, routine immunization, supplementary immunization activities, vaccination, vaccines

The elimination of poliovirus from nearly all countries is one of the major public health successes of the modern era. At the start of the Global Polio Eradication Initiative (GPEI) in 1988, there were more than 350,000 cases of paralytic polio reported globally; by 2023, just 12 cases of wild poliovirus (WPV) were confirmed globally (1, 2). Furthermore, two of three serotypes of wild poliovirus have been eradicated, leaving only wild poliovirus type 1 (WPV1) as the only circulating wild serotype of the virus in just two countries, Pakistan and Afghanistan (1, 3). These countries have never interrupted endemic transmission of WPV1; however, both have made considerable progress toward the goal of polio eradication. In Pakistan, significant human and financial resources have been committed to polio eradication activities, with an extensive network of acute flaccid paralysis (AFP) and environmental surveillance sites enabling tracking and detection of areas with ongoing poliovirus circulation (4).

Additionally, supplementary immunization activities (SIAs) are being implemented in the country several times a year, nationally and subnationally, to bolster polio vaccination coverage amongst children < 5 years of age, prioritizing areas with weak healthcare systems and suboptimal routine immunization coverage (4, 5). These vaccination activities, typically implemented using oral poliovirus vaccine (OPV) and aligned with the GPEI strategic plan (6), have accounted for significant reductions in the numbers of children with paralytic polio in Pakistan, but considerable obstacles to eradicating WPV1 remain. This research article collection spotlights the impediments to eradicating polio in Pakistan and what is being done to surmount them while highlighting an outbreak of circulating vaccine-derived poliovirus type 2 (cVDPV2) elsewhere in the Middle East and the concomitant threat posed to the broader goal of a polio-free world.

The Research Topic “*Eradicating polio in Pakistan and other countries in the Eastern Mediterranean region: epidemiology, surveillance, and immunization*” received a total of 14 submissions, of which 10 were accepted and 4 were rejected. Of the 10 articles accepted and published in the Research Topic, seven were original research studies; there was one community case study, one data report, and one opinion article. A total of 73 authors contributed to the published articles, reaching a broad scientific audience encompassing many countries drawn from different continents around the globe. This underscores the continuing relevance and importance of polio eradication efforts in the larger scheme of global health security.

Larik et al. describe how a resurgence of WPV1 cases in 2024 followed a decline in the quality of polio vaccination campaigns in Quetta block, Balochistan province, Pakistan, in the preceding years. Community boycotts and insecurity factored in the decline in the quality of vaccination activities in Quetta block, as was the case in south Khyber Pakhtunkhwa, Pakistan, where similar challenges in implementing polio SIAs and the strategies adopted to overcome them are the focus of a paper by Khan et al. and Ben Hamida et al. discuss the findings of a study intended to estimate the proportions of and reasons for missed children following a special immunization activity aimed at reaching children under 5 years of age in 69 high-risk union councils of south Khyber Pakhtunkhwa, where operational reasons accounted for three times as many missed children as refusals.

Community engagement and the use of conditional incentives to enhance polio vaccine uptake in high-risk union councils of Bannu and Karachi, Pakistan, constitute the subject of an original research study by Tabassum et al. A similar study by Abbasi et al. focuses on how integration of vaccination services into the delivery of other medical services, such as maternal and child healthcare, administered through health camps improved the uptake of polio vaccines in Karachi, Sindh province, Pakistan. The value of these services in reaching underserved communities in a setting of conflict and humanitarian crises is the subject of an opinion article by Menon et al. on the implications of the detection of vaccine-derived poliovirus type 2 (VDPV2) in Gaza's sewage system.

Eradicating polio requires not only vaccination but also the rapid detection of circulating poliovirus through the AFP surveillance system. Effectiveness of the surveillance system depends on the knowledge and responsiveness of frontline health workers. A study by Geiger et al. discusses how stool specimen inadequacy, often linked to delayed notification of AFP cases, exposed knowledge gaps amongst health workers on AFP surveillance, the system upon which poliovirus detection is contingent. The risks posed by such deficiencies in the surveillance system are highlighted in an article by Saleh et al. which shared the findings of a 2024 rapid self-assessment among health workers in Gaza, showing that they felt under-equipped to detect and respond to polio outbreaks in the context of VDPV2 circulation in Gaza. Also on the Gaza polio outbreak, the knowledge, attitudes, and practices of mothers attending vaccination sites constitutes the topic of a study by Bilbeisi et al. demonstrating a discordance between good knowledge and positive attitudes toward vaccination and lower levels of adherence to good vaccination practices.

The emergence of cVDPV2 in the wider region is a parallel and increasing threat. The biological basis for this threat is quantified in a seroprevalence study by Hussain et al. that assessed the change in poliovirus type 2 immunity in 12 high-risk areas of Pakistan following the synchronized withdrawal of trivalent OPV (tOPV) from the immunization schedules of all countries (7). The study showed considerably lower seroprevalence of immunity to type 2 poliovirus (< 50%) compared with immunity to type 1 and type 3 poliovirus (>90%). By implication, immunity to type 2 poliovirus has waned since the cessation of tOPV, leading to the emergence of cVDPV2 outbreaks in countries with weak immunization systems (8). However, the introduction of inactivated poliovirus vaccine (IPV) into the routine immunization systems of countries worldwide (7), including Pakistan, has mitigated the risk of these outbreaks, where high levels of coverage have been achieved. Concurrent administration of monovalent OPV type 2 (mOPV2) vaccine or the more genetically stable novel OPV type 2 (nOPV2) vaccine (9) during outbreak response SIAs has helped to improve the poliovirus type 2 immunity profiles of children in areas with suboptimal IPV coverage.

While mitigating the risk of cVDPV2 emergence continues to be a priority for Pakistan and other countries in the Middle East, the focus in Pakistan remains on the goal of interrupting endemic transmission of WPV1. To achieve this, renewed and consistent efforts must be placed on finding innovative ways to engage hesitant communities to increase polio vaccine acceptance and uptake while ensuring that high levels of coverage are maintained in areas where there are no barriers to access for the polio program.
